# Abortion, and Menstruation during Pregnancy

**Published:** 1848-02

**Authors:** 


10. *Abortion, and Menstruation during Pregnancy.*—[Mr. Whitehead, in a recent work on the Physiological and Morbid Conditions of the Uterus and their relations to the Treatment of Abortion and Sterility, when treating of Abortion, lays down the three following positions from the cases narrated:]

1. That what is commonly called ulceration of the cervix uteri may be the predisposing, as well as the immediately exciting cause of abortion.

2. That the purulent product of uterine ulceration, under some forms, at least, possesses virulent properties, capable of producing disease in another individual, or in another part of the same individual by inoculation; and probably capable also, by being absorbed into the circulation of the same person, of materially disordering the fluids, and of creating thereby a peculiar susceptibility to disease.

3. That the application of caustic to the uterus, and the employment of other active measures which I have heard practitioners object to during pregnancy, as likely to endanger the well-being of the offspring, may not only be safely administered, but that they constitute in fact one of the principal means of securing both mother and child from danger.

[In relation to menstruation during pregnancy, the following are his conclusions:]

1. That menstruation during pregnancy is, for the most part, perhaps always, associated with an abnormal condition, generally with ulcerative disease of the uterus, requiring at all times active remedial treatment.

2. That hemorrhage during pregnancy is not necessarily associated with an altered relation of the parts within the uterus, and, by timely care, need not interfere with the integrity of the ovum.

3. That menstruation, during the early periods of lactation, is not always natural menstruation, but that it is generally associated with morbid conditions which are amply adequate to the satisfactory explanation of the phenomenon; that secondary hemorrhage is, in the majority of instances, not owing to imperfect contraction, or atony of the uterine fibres; and that the discharge very probably proceeds, under these circumstances, not from the inner surface of the uterus, but from the diseased surfaces, situated upon parts external to the cavity of the organ.—*Ibid.*

